# Image-Guided Robotic Radiosurgery for the Treatment of Same Site Spinal Metastasis Recurrences

**DOI:** 10.3389/fonc.2021.642314

**Published:** 2021-05-28

**Authors:** Felix Ehret, Lucas Mose, Markus Kufeld, Christoph Fürweger, Paul Windisch, Alfred Haidenberger, Christian Schichor, Jörg-Christian Tonn, Alexander Muacevic

**Affiliations:** ^1^ Charité—Universitätsmedizin Berlin, Corporate Member of Freie Universität Berlin and Humboldt-Universität zu Berlin, Department of Radiation Oncology, Berlin, Germany; ^2^ European Cyberknife Center, Munich, Germany; ^3^ Department of Stereotaxy and Functional Neurosurgery, University Hospital Cologne, Cologne, Germany; ^4^ Department of Radiation Oncology, Kantonsspital Winterthur, Winterthur, Switzerland; ^5^ Department of Neurosurgery, Ludwig-Maximilians-University Munich, Munich, Germany

**Keywords:** SBRT, radiosurgery, spinal metastasis, spine, recurrence, CyberKnife, reirradiation (ReRT)

## Abstract

**Background:**

Due to recent medical advancements, patients suffering from metastatic spinal disease have a prolonged life expectancy than several decades ago, and some will eventually experience relapses. Data for the retreatment of spinal metastasis recurrences occurring at the very same macroscopic spot as the initially treated lesion are limited. Previous studies mainly included recurrences in the boundary areas as well as other macroscopic parts of the initially affected vertebrae. This study exclusively analyzes the efficacy and safety of spinal reirradiation for recurrences on the same site utilizing single-session robotic radiosurgery.

**Materials and Methods:**

Patients between 2005 and 2020 who received radiotherapy for a spinal metastasis suffering from a local recurrence were eligible for analysis. Only patients undergoing a single-session reirradiation were included. All recurrences must have been occurred in the same location as the initial lesion. This was defined as a macroscopic recurrence on computed tomography occurring at the same site as the initial spinal metastasis. All other lesions, including those in the boundary areas or other parts of the initially affected vertebrae, were excluded.

**Results:**

Fifty-three patients with fifty-three lesions were retreated for spinal metastases. The median dose and number of fractions for the initial radiotherapy were 36 Gy and 15, respectively. Eleven patients were initially treated with stereotactic body radiotherapy. Retreatment was performed with a median dose of 18 Gy prescribed to a median isodose of 70%. The local control was 77% after a median follow-up of 22.2 months. Patients experiencing a second recurrence received a lower dose (p = 0.04), mostly below 18 Gy, and had a worse coverage (p = 0.01) than those showing local tumor control. 51% of patients experienced an improvement in pain control after treatment delivery. Besides, four vertebral compression fractures (7% of patients) but no other adverse events higher than grade 2 were observed.

**Conclusion:**

Single-session robotic radiosurgery appears to be a safe, time-saving, and effective treatment modality for spinal metastasis recurrences occurring in the same initial location if a considerable dose and coverage can be applied. Treatment results are comparable to reirradiated metastases in the boundary areas.

## Introduction

Due to recent medical advancements, patients suffering from metastatic disease have a prolonged life expectancy than several decades ago (1). In addition to projected demographic changes, this shift is expected to lead to an increasing number of patients needing therapy for spinal metastases within the upcoming years. Today, about 180.000 patients in the United States are suffering from spinal metastases, and approximately 10% of them will experience spinal cord compression as a potentially life-threatening complication (2–5). With the development, implementation, and general availability of conventional fractionated external-beam radiotherapy (EBRT) and stereotactic body radiation therapy (SBRT), many patients with spinal metastases can be treated effectively and non-invasively. Primary treatment results regarding local control (LC), pain, and quality of life (Qol) are sound (6, 7). However, with the increased life expectancy after irradiation, chances of local recurrence or even further development of spinal metastases increase. So far, single-session and multisession SBRT up to 5 fractions showed 1-year-LC rates around 80% for spinal metastases, even for radioresistant tumor entities (6, 8–11). Still, this implies that a substantial number of patients will experience the need for a follow-up treatment thanks to current and future improvements in systemic therapies, which increase the overall life expectancy for patients with metastatic disease. Thus, dedicated treatment options for recurrent spinal metastases are needed. These should respect the previous irradiation and associated risks for myelopathies and vertebral compression fractures (VCFs) (12). Reirradiation for spinal metastases seems feasible and effective, but the number of reports is still limited (12–15). Finally, data on the efficacy and safety of reirradiation with single-session robotic radiosurgery (RRS) are particularly limited (12, 14). Besides, previous reports often included spinal recurrences in the boundary area or previously irradiated field and other parts of the initially affected vertebrae, and not just same site relapses (16, 17). This may potentially compromise patient and data homogeneity, which could limit the overall generalizability of the reported results. Thus, the objective of this study is to analyze the treatment results of RRS for preirradiated spinal metastasis recurrences occurring at the very same spot as the initial macroscopic lesion. To date, data for this specific patient cohort are sparse. Besides, all tumors were exclusively and primarily treated in one session, and we compare our results with the existing SBRT literature.

## Materials and Methods

Fifty-three patients treated for a spinal metastasis recurrence between 2005 and 2020 were included in this retrospective single-center study. Only patients undergoing primary single-session RRS for a retreatment for relapse on the same site as the initial tumor were eligible for analysis. This type of lesion was defined as a macroscopic osteolytic, osteoblastic, or mixed recurrence on computed tomography (CT) occurring at the same site as the initial macroscopic spinal metastasis. Patients undergoing initial surgical treatment for their relapse before RRS and local recurrences in the boundary area of the previous irradiation as well as other parts of the affected vertebrae were excluded. All patient data, including medical history, previous treatments, and follow-up data, were prospectively stored in a dedicated database for radiosurgery and retrospectively analyzed. Diagnosis of spinal metastasis recurrence was made by an interdisciplinary team consisting of radiation oncologists, neurosurgeons, and neuroradiologists.

### Treatment Procedure and Outcome

As formerly described, every patient underwent thin-sliced, contrast-enhanced CT and magnetic resonance imaging (MRI) scans for treatment planning and delivery. Obtained images were overlaid for inverse treatment planning, which was done with a dedicated planning software (MultiPlan, Precision, Accuray Inc., Sunnyvale, CA, USA). All treatments were delivered in a single session utilizing a CyberKnife^®^ robotic radiosurgery system (Accuray Inc., Sunnyvale, CA, USA). For tracking, the X-sight spine tracking algorithm (Accuray Inc., Sunnyvale, CA, USA) has been used for all treatment sessions without any application of fiducials (18, 19). The gross tumor volume (GTV) comprised all visible tumor tissue identified on MRI and CT scans. For vertebral body metastases, a 2 mm margin was added. Besides, no further margins have been added to the planning target volume (PTV), given the accuracy of RRS (19). Radioresistant tumors included renal cell carcinoma, gastrointestinal tumors, non-small cell lung cancer, sarcomas, head and neck tumors, thyroid cancer as well as melanomas. Radiosensitive tumors included breast, cervical, uterine, and prostate cancer. The metastases of cancers of unknown primary were deemed intermediate. This classification was in accordance with the work of Yamada et al. (20). Dose constraints for organs at risk were respected for patients following the data of the AAPM TG101 if medically appropriate and feasible as well as subject to changes for individual cases (21). Dose constraints were as follows: ≤0.35/≤1.2 cc of the spinal cord could receive 10.0/7.0 Gy, with a maximum point dose of 14.0 Gy in ≤0.35 cc. Adverse events (AE) and toxicity were reported according to the Common Terminology Criteria for Adverse Events (CTCAE) up to version 5, depending on the date of the AE occurrence. Local control (LC) was defined as an unchanged or decreased tumor volume on follow-up imaging. Local failure (LF) was defined as an increased tumor volume during follow-up.

### Statistical Analyses

Time of LC, local progression-free survival (including LFs and death of any cause) (l-PFS), and overall survival (OS) were calculated according to the Kaplan-Meier estimate. Differences in survival or other time-to-event differences were analyzed with a log-rank test. Continuous variables were tested for normality utilizing the Shapiro Wilk test and the graphic appearance, including skewness and kurtosis. Subsequent analyses were done with unpaired t-tests or Wilcoxon rank-sum tests according to the results of the normality testing, For categorical testing, the Fisher’s exact test was utilized if the number of events was less than five in each group; for scenarios with more than five events for each group, the Chi-square test was applied. Descriptive statistics utilized mean, median, frequencies, proportions, and ranges depending on the analyzed variable. Data were analyzed using STATA 16.0 MP (StataCorp, College Station, TX, USA). P-values equal to or less than 0.05 were considered significant.

## Results

### Patients and Treatment Characteristics

Patient, pretreatment, and treatment characteristics are summarized in **Tables 1** and **2**. A total of 53 patients with a median follow-up of 22.2 months were included in this analysis. The initial treatment was mainly fractionated (41/53 patients, 77.3%), with a median dose of 36 Gy. The median time to recurrence was 17.2 months. A total of five patients (9%) experienced a recurrence within six months of upfront treatment, three of them after three months. Four of them received EBRT; one underwent SBRT. For the recurrences, a median dose of 18 Gy prescribed to a median isodose of 70% was applied. The median and mean max doses to the spinal cord were 13.4 and 13.7 Gy, respectively. Most of the lesions were osteolytic, located in the lumbar spine, and caused pain. The majority of treated entities were deemed radioresistant. The renal cell carcinoma was the most frequently treated tumor in this cohort. Forty patients had further metastatic disease; thirteen were only suffering from the spinal metastasis recurrence. Only four patients (7%) had brain metastases at the time of treatment delivery. Most patients were suffering from further bone metastases (58%), abdominal metastases (34%), and lung metastases (11%). Four patients (7%) suffered from VCFs after reirradiation. Besides, the reirradiation was well tolerated; no patients experienced other AE grade 2 or higher. No myelopathy or bleeding events were observed after retreatment.

**Table 1:** Patient characteristics.

Total number of patients included		53		
Gender (male/female, %)	34 (64)	19 (36)		
	**Median**	**Mean**	**Range**
Age (years)	61.9	62.5	36.3 – 89.4
Pretreatment Karnofsky Performance Status (%)	90	91.8	70 – 100
Follow-up (months)	22.2	34.7	1.4 – 154.3
**Tumor location**	**Cervical**	**Thoracic**	**Lumbar**
Number of patients	11	19	23
%	21	36	43
**Lesion type**	**Osteolytic**	**Osteoblastic**	**Mixed**
Number of patients	32	20	1
%	60	38	2
**Symptoms**	**Pain**	**Radiculopathy**	**Weakness**
Number of patients	21	7	2
%	40	13	4
**Only spinal metastasis, no other distant metastasis**	**Yes**	**No**	
Number of patients	13	40	
%	23	77	
**Tumor entities**		**Number of patients**	
Renal		14
Breast		10
Prostate		8
Lung		7
Head and neck		2
Colorectal		2
Other		10
**Radiosensitivity**	**Sensitive**	**Intermediate**	**Resistant**
Number of patients	18	2	33

**Table 2:** Pretreatment and treatment characteristics.

Pretreatment	Median	Mean	Range
Dose (Gy)	36	33	14 – 50.4
Number of fractions	15	13.9	1 – 28
Time to recurrence (months)	17.2	27.9	2.5 – 236
Patients with a time to recurrence of less than six months (%)		5 (9)
Patients treated with one fraction (%)		12 (23)
Patients treated with two to five fractions (%)		3 (6)
Patients additionally treated with surgery (%)		5 (9)
**Treatment**	**Median**	**Mean**	**Range**
Tumor volume (cc)	25.7	35.5	1.5 – 115.5
Prescription dose (Gy)	18	18.7	15 – 22
Prescription isodose (%)	70	68.3	60 – 75
Max tumor dose (Gy)	27.1	27.4	20.7 – 34.5
Min tumor dose (Gy)	11.7	12.5	8.3 – 20.3
Max dose spinal cord (Gy)	13.4	13.7	1.8 – 21.8
Conformity index	1.28	1.31	1.13 – 1.74
Homogeneity index	1.43	1.47	1.33 – 1.67
Coverage	93.7	92.5	76.6 – 99.9

cc, cubic centimeter; Gy, Gray.

### Local Outcome and Survival Data

**Figure 1:**
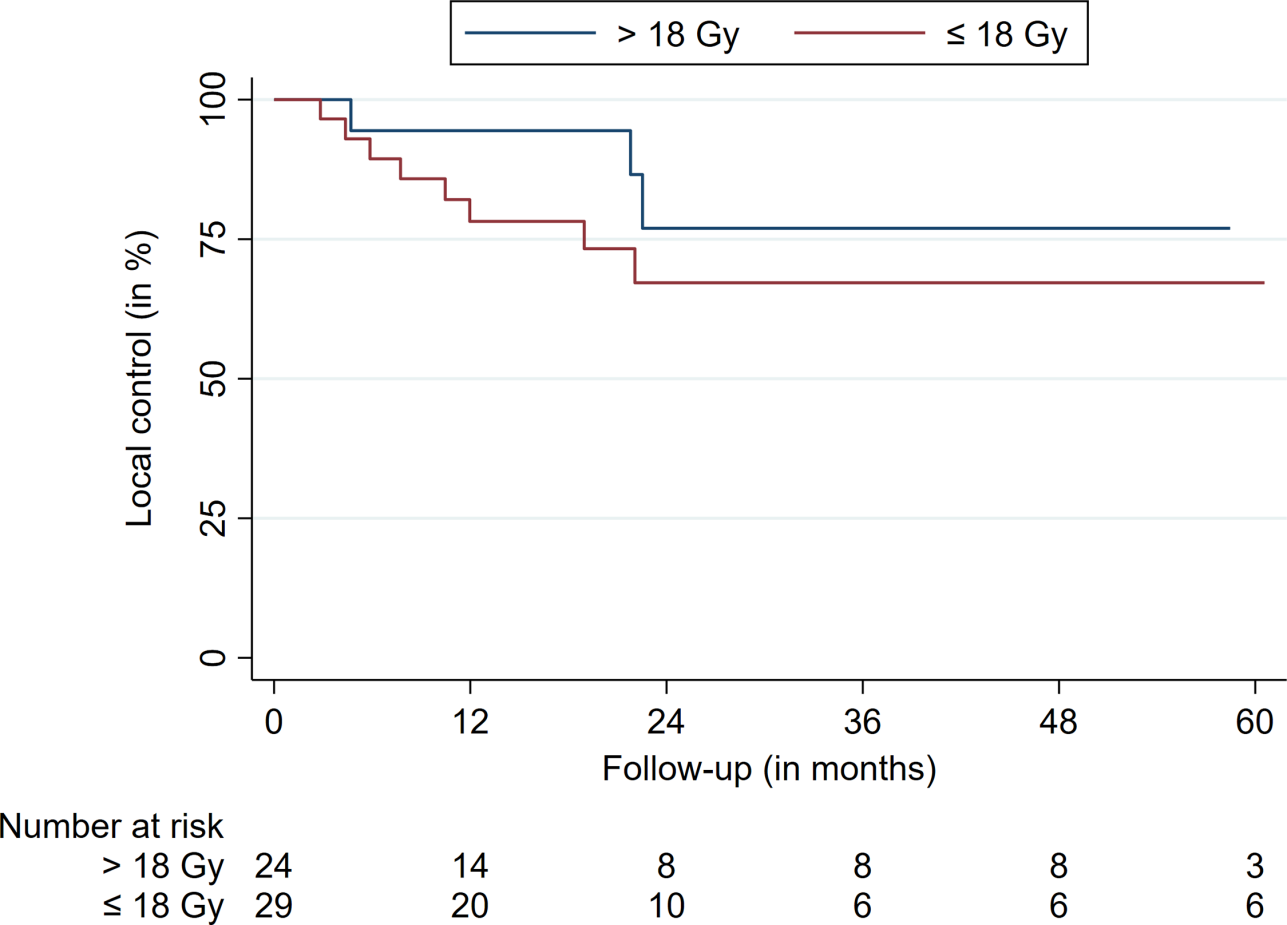
Local control stratified for a prescription dose cutoff at 18 Gy.

The comparison between locally-controlled and uncontrolled patients is summarized in **Table 3**. The outcome and survival data are outlined in **Table 4**. Overall, 12 of 53 patients experienced a second recurrence of their spinal metastasis after a median and mean time of 14.7 and 22.7 months, respectively. This equals a crude LC of 77.3%. Overall, the prescription dose and coverage were lower in patients suffering from TF (p = 0.04 and p = 0.01, respectively). The minimal dose within the tumor showed a trend but did not reach significance (p = 0.07). Most of the recurrences occurred in patients with prescription doses of 18 or less Gy (p = 0.04, 9/12 patients, 75%) (**Figure 1**). Besides, most LFs were present in patients with a coverage less than 94% (p = 0.01, 9/12 patients, 75%).

**Table 3:** Comparison between locally-controlled and uncontrolled patients.

Variable	Local control	Treatment failure	p-value
	Mean (±SD)	
Age	61.3 (2.3)	62.7 (2.8)	0.76
Time to first recurrence (months)	28.9 (6.1)	24.5 (5.6)	0.94
Pretreatment fractions (number)	13.0 (1.4)	16.8 (2.4)	0.20
Pretreatment dose (Gy)	32.2 (1.5)	35.7 (3.0)	0.30
Tumor volume (cc)	36.3 (5.0)	38.6 (7.7)	0.82
Dose (Gy)	18.9 (0.2)	17.9 (0.5)	0.04
Max dose (Gy)	27.7 (0.4)	26.6 (0.9)	0.13
Min dose (Gy)	12.9 (0.5)	11.2 (0.4)	0.07
Coverage (%)	93.6 (0.6)	89.5 (1.9)	0.01

SD, standard deviation; Gy, Gray; cc, cubic centimeter.

**Table 4:** Outcome and survival data.

Variable	Time (in months)	Value (%)	95% Confidence interval (%)
**LC**	12	85.1	71.4 – 92.6
	24	72.9	55.9 – 84.2
	36	72.9	55.9 – 84.2
**l-PFS**	12	72.2	57.7 – 82.5
	24	47.0	32.1 – 60.5
	36	36.6	22.5 – 50.7
**OS**	12	82.0	68.2 – 90.2
	24	58.9	43.2 – 71.6
	36	43.1	28.0 – 57.4

LC, local control; l-PFS, local progression-free survival; OS, overall survival.

**Figure 2:**
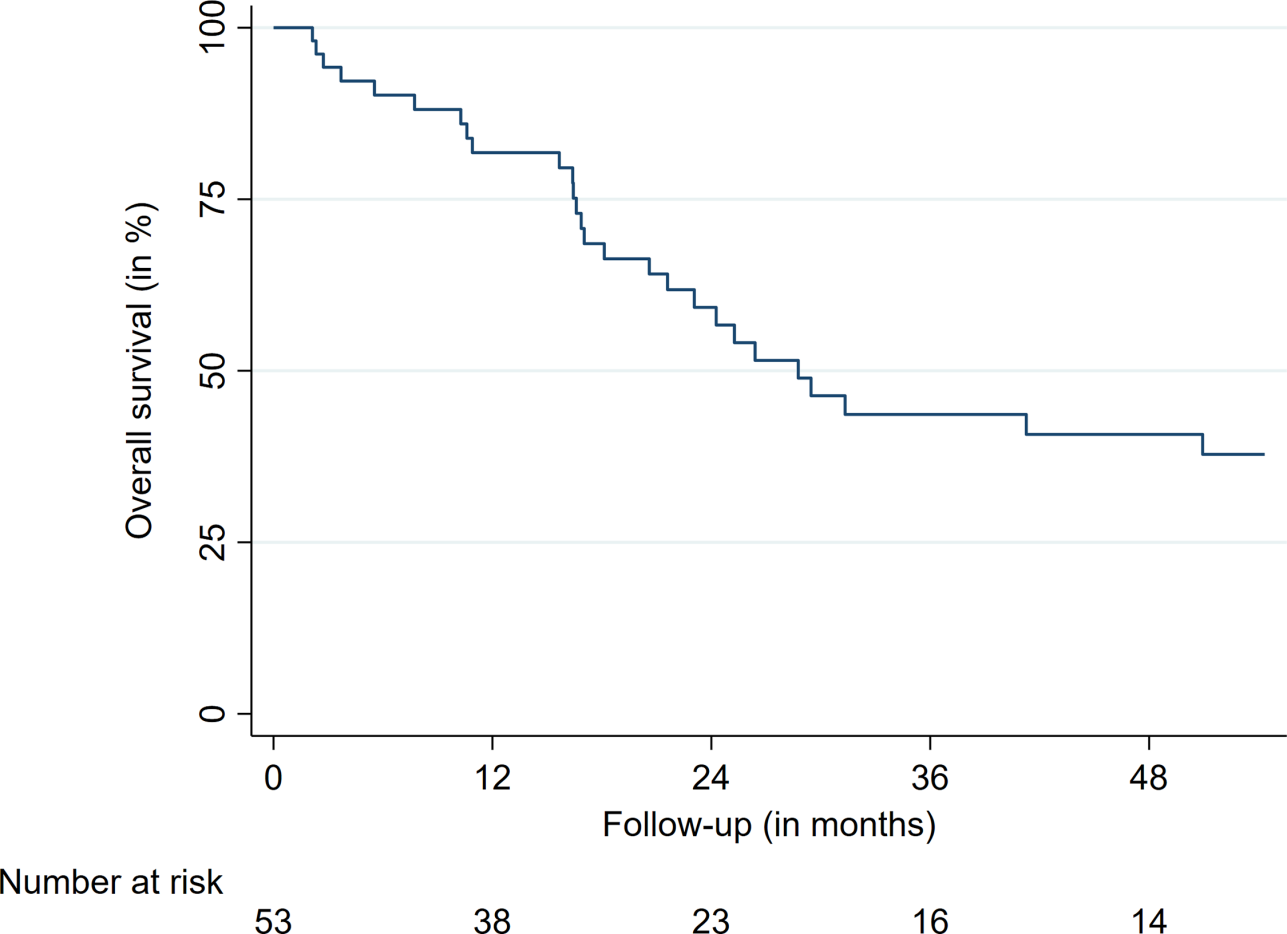
Overall survival.

**Figure 3:**
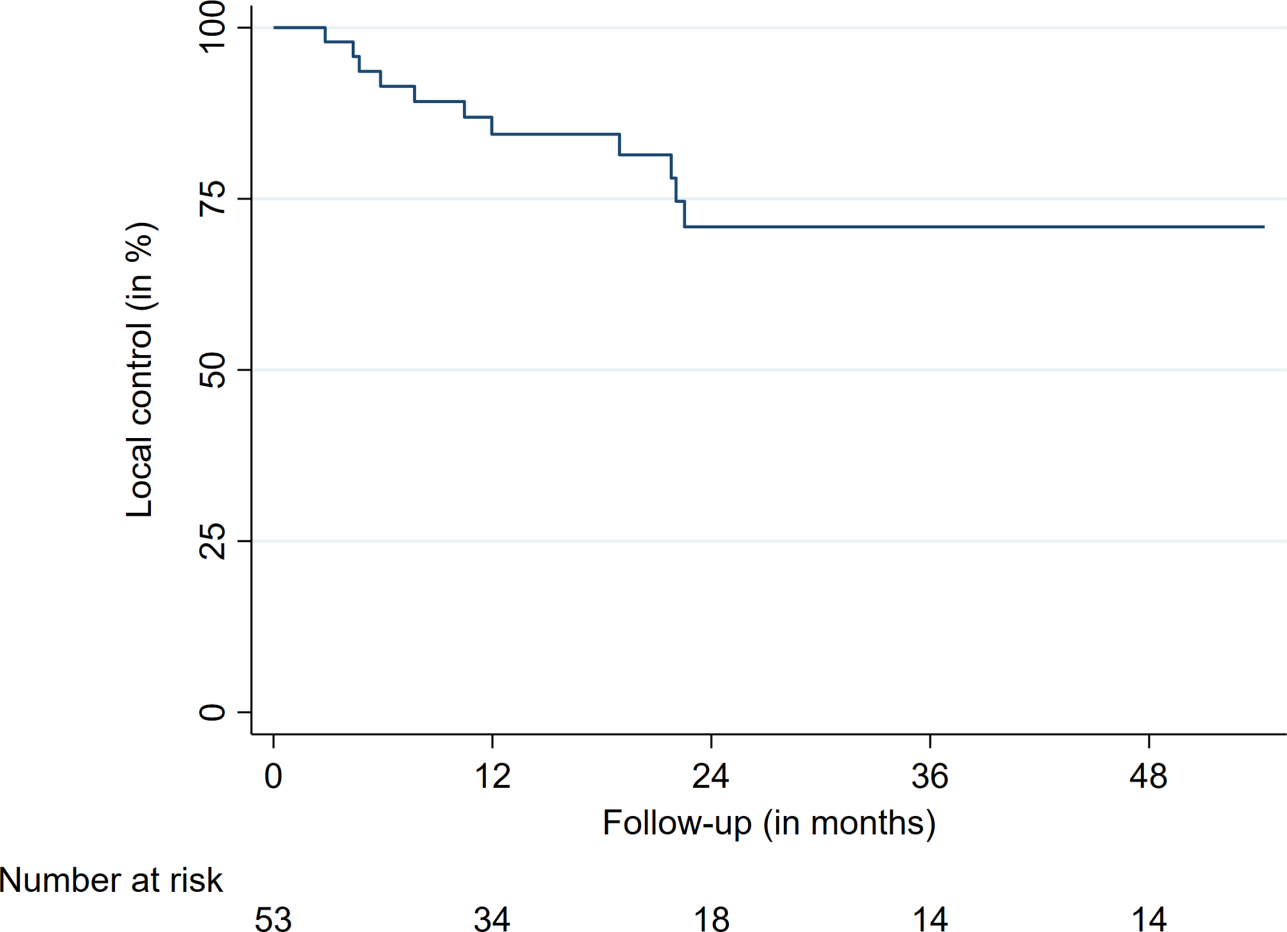
Overall local control.

**Figure 4:**
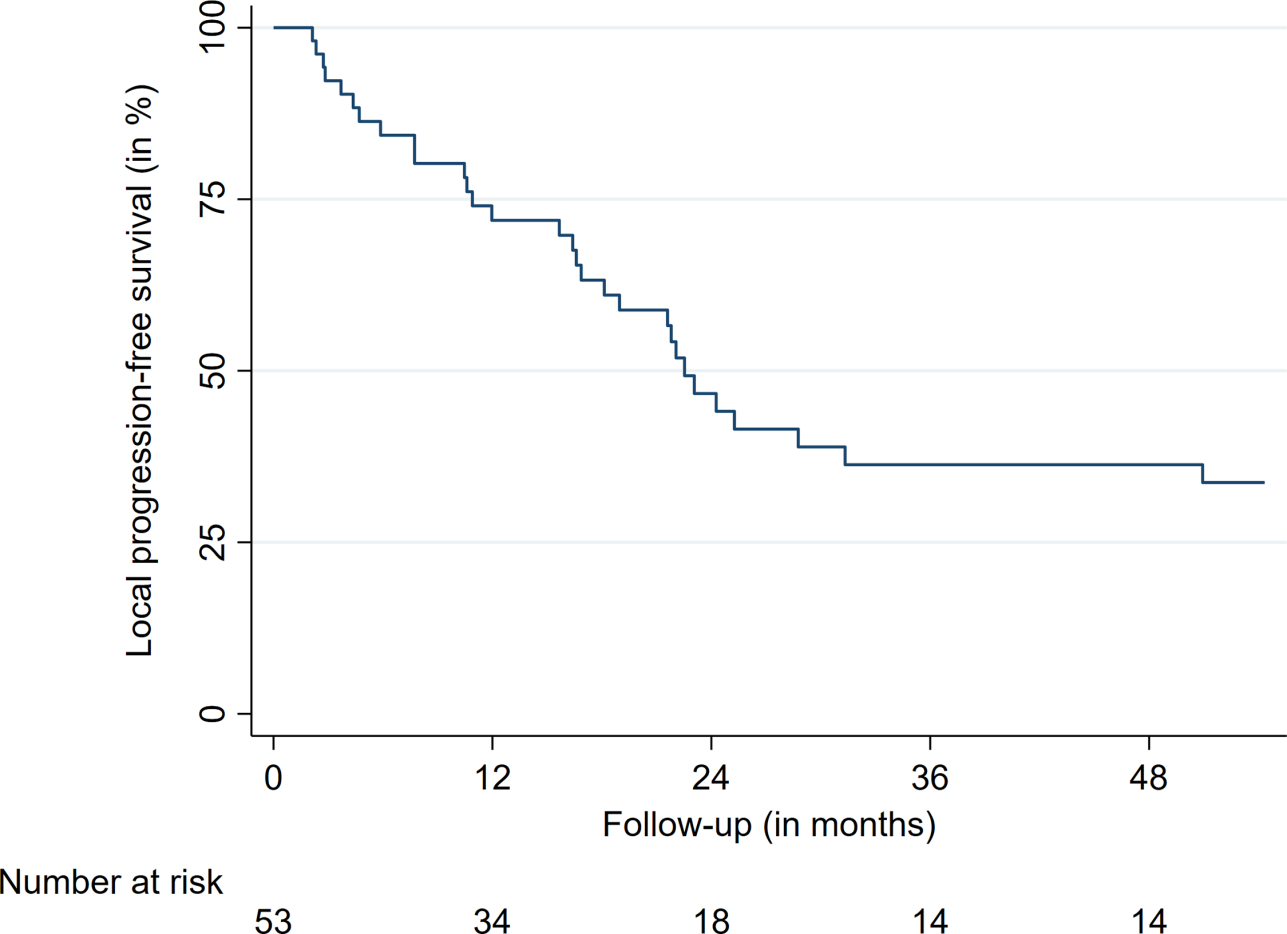
Local progression-free survival.

The median survival was 28.7 months (**Figure 2**). The LC after 12, 24, and 36 months was 85.1%, 72.9%, and 72.9%, respectively (**Table 4**and **Figure 3**). The respective l-PFS and OS were 72.2%, 47.0%, 36.6% and 82.0%, 58.9%, and 43.1% (**Table 4**, **Figures 2** and **4**). The OS did not significantly differ for patients receiving more than 35 Gy or less for their first treatment (p = 0.15). LC rates did not differ for patients receiving their reirradiation within or after 12 months of initial treatment delivery (p = 0.90). Radiosensitivity did not have a significant impact on LFs. For 29 patients, clinical status was obtainable at last follow-up, with 51% of patients experiencing a subjective improvement in pain control. Moreover, 20% had a stable pain level and 29% showed worsening of their subjective pain at their last follow-up. Notably, most patients in this study experienced a significant decrease in their overall performance status and progressed due to metastatic disease, partially limiting dedicated clinical pain evaluations regarding the preirradiated spinal metastasis. At last follow-up, thirty-six patients (68%) had disease progression or succumbed to their illness.

## Discussion

This is one of the largest reports exclusively analyzing patients treated with single-session RRS for their spinal metastasis recurrence (12–14). Only recurrences which occurred at the very same spot as the initial macroscopic metastatic lesion were included. So far, only sparse data are available for this patient cohort. The objective was to investigate whether this dedicated subgroup of recurrences behaves differently when irradiated with SBRT. Besides, the reported follow-up herein is more extensive compared to most of the previous studies (12, 13). In general, spinal metastases are a common and considerable oncologic challenge. With the recent advancements in systemic and local treatments, more patients will experience spinal metastasis recurrences in the foreseeable future. However, only a few studies have reported dedicated results for SBRT for the treatment of spinal metastasis recurrences until today (12, 13). Thus, further evaluation of treatment options that may achieve long-term LC and pain relief are needed.

### Local Control and Survival

According to recent reviews, SBRT for the reirradiation of spinal metastasis has shown 1-year LC rates between 66% and 90% (12, 14). Overall, the current data quality on spinal reirradiation with SBRT for spinal metastasis are not only limited but mostly based on retrospective single-center trials like the current study. One large retrospective multicenter analysis showed favorable results for single-session treatments (13). Notably, most reports included recurrences that generally occurred in previously irradiated fields, while this study exclusively included recurrences literally at the same spot as the initial lesion, trying to improve data homogeneity. Our findings, however, are mostly comparable to the previous reports (12–14, 23). Moreover, this report only included patients receiving one fraction. Many preirradiated patients in the literature underwent more than one fraction. The most common fractionation schemes included 3 x 9 Gy, 3 x 8 Gy, 3 x 7 Gy, 5 x 6 Gy, 5 x 5 Gy, and 5 x 4 Gy, with comparable LC rates (12–14). Notably, a single-session treatment reduces the time patients need to spend for their treatment and care. This is especially important for patients receiving palliative care. As previously described, 1-year LC rates are around 80% with the formerly mentioned doses and fractions. In this study, 85% of patients had their metastasis controlled after 12 months. This is also in agreement with the patients treated with one fraction, as reported by Hashmi et al. (13). Notably, they reported better LC rates for single-session treatments compared to multisession irradiations (13). As most published series only report 1-year LC rates due to poor overall survival of the study cohorts, respective median follow-up times are mostly around 12 months (12, 13). Thus, not much data are available on the LC beyond this period after undergoing SBRT. Herein, many patients were alive after two years, showing a LC of 73% at that time. After three years, 16 patients were still alive, with a LC rate of 73%. Despite limited data and the small sample sizes, SBRT may achieve satisfactory 2-year and 3-year LC rates (13). However, it remains unclear what factors may influence LC rates in this patient group. In contrast to Garg and colleagues as well as Choi and colleagues, we did not find significant associations between pretreatment doses of less than 35 Gy or the time to reirradiation less than 12 months with OS and LF (16, 24). Overall, it remains unclear to which extent the primary treatment influences the outcome of the reirradiation. Nevertheless, we did see the trend that patients receiving lower prescription doses (<18 Gy) experienced most of the documented LFs. Considering that doses around 24 Gy delivered in one fraction show reasonable LC rates for the initial treatment of spinal metastasis, it may decrease the chance of LF if doses of at least 19 Gy in one fraction may be applied (25). Besides, patients with a minimal dose of 13 Gy or less experienced significantly more local recurrences. Moreover, patients with a coverage of more than 94% did show fewer recurrences. Whereas previous studies and reports discussed the role of radiosensitivity as a potential factor influencing the LC rate, we did not find any significant associations herein (20, 26). However, this may be due to the small sample size, low number of LFs, and proportions of included tumor entities.

Despite the fear of associated toxicity and adverse events with a limited life expectancy, treating physicians should anticipate increased survival in this patient subgroup in the future and, thus, should try to apply a considerable dose with respective coverage to prevent LFs before systemic disease progression. Overall, treatment planning should be carefully evaluated for this specific patient subgroup. This is especially important for patients only suffering from spinal metastasis while having a controlled primary tumor side and a low systemic tumor burden. Finally, the applied single-session RRS treatment achieved LC in most of the cases, but the majority of patients suffered from additional metastases at the time of treatment, which ultimately led to an overall disease progression. Finally, most other reports did not report on the further disease status of patients, i.e., presence and number, as well as the location of other metastases, limiting accurate comparisons.

### Toxicity

Spinal reirradiation with SBRT at the same macroscopic location is still not a very commonly reported situation. In this study, we observed tolerable toxicity, mostly following the dose constraints of the AAPM TG 101 (21). No adverse events higher than grade 2 were observed after treatment delivery. The four occurring VCFs except one did not need any additional medical or surgical treatment. Previously published studies reported similar toxicities, with the majority of AE related to fatigue. An overall VCF rate of 12% was reported among the four studies reporting VCFs as a dedicated adverse event in a recent review (12). With a VCF rate of 7%, single-session RRS seems to have a slightly lower fracture risk as compared to the hypofractionated schemes that were applied in the other studies. However, in contrast to the VCF rate of 4.5% in the study of Hashmi et al., the risk seems to be slightly higher (13). Yet, data are limited, and no definite conclusions can be drawn considering that the majority of studies did not report on the occurrence of VCF (12, 14). Besides VCF, myelopathy is an associated complication after spinal irradiation. In this study, no myelopathies were observed in our group of 53 patients. This is in agreement with the existing literature as myelopathies still rarely occur after reirradiation (crude risk 1.2%) (12, 13). Overall, and given the limited data available for modern SBRT for spinal metastasis reirradiation, it remains unclear what other factors may contribute to adverse events higher than grade 2.

### Pain

Pain control has always been a substantial treatment goal for the treatment of spinal metastases. This goal is similar for the retreatment of spinal lesions. While repeated EBRT with various fractionation schemes showed a notable effect in around 60% of patients in multiple studies, some data are available on SBRT for the control of pain after reirradiation (12, 22, 27). Moreover, given the heterogeneity of reporting outcomes for spinal treatments – something which is especially true for reirradiation procedures –, limited conclusions can be drawn from the available data (12–14, 28). Current studies show pain control rates between 65% and 81% (12, 13). As for the dynamic of pain control and improvement, Garg and colleagues reported better pain levels on the Brief Pain Inventory (BPI) after three months of treatment delivery (24). This improvement was also present after six months (24). Herein, we report an improvement rate of 51% at last follow-up, which may be caused by the limited clinical information and subjective assessment method. Considering the limited data we can provide on pain control, more studies are needed to assess the actual treatment efficiency. Notably, most cases in this report had widespread metastatic disease, especially bone metastases, with respective symptoms that limited the possibility to exactly determine the symptoms just caused by the spinal recurrence alone.

### Future Challenges

As depicted by the SPINO consortium, the reporting of studies for spinal metastasis is particularly heterogeneous (28). Considering the 15-year span it took to treat about 50 patients with a spinal recurrence occurring in the exact location as previously, the frequency of spinal reirradiation for this small subgroup of patients poses a considerable challenge to report standardized outcomes. This is mainly due to various changes in the field and improved radiation techniques in the past 15 years. With respect to the increasing life expectancy of patients with spinal metastasis, patients experiencing recurrences should be assessed in a comparable way to create reliable evidence on reirradiation treatment options. According to the SPINO consortium, various clinician-based (SINS, Bilsky grade, MRC, KPS,…) and patient-reported outcomes (SF36, BPI, SOSGOQ,…) should be implemented (28). Besides these recommendations and in consideration of the available literature, single-session RRS may be an appropriate tool for spinal reirradiation, especially in palliative settings. Ultimately, prospective trials are necessary to determine the ideal management on spinal reirradiation.

### Limitations

This study has several inherent limitations given its retrospective nature and design. First, the sample size is limited due to the single-center study design. Second, no standardized outcome measures for the pain assessment were available. The analysis of the pain data was limited to chart reviews. Third, we included all patients who met the criteria for their recurrence, potentially causing a sampling bias due to this convenient sampling approach. This may be reflected by an imbalance of patients receiving EBRT and SBRT as their initial treatment.

## Conclusion

Single-session RRS appears to be a safe and effective treatment modality for spinal metastases reoccurring at the same macroscopic location after initial irradiation. Treatment results are comparable to reirradiated metastases in the boundary areas. Toxicity can be effectively limited if appropriate dose constraints are considered. Given the practicability, single-session RRS may be a well-suited treatment option given the less time-consuming treatment delivery if reasonable doses with an adequate coverage can be applied.

## Data Availability Statement

The data that support the findings of this study are available from the corresponding author, FE, upon reasonable request.

## Ethics Statement

The studies involving human participants were reviewed and approved by the Ludwig-Maximilians-University Munich. Written informed consent for participation was not required for this study in accordance with the national legislation and the institutional requirements.

## Author Contributions

Conception and design of the study: FE. Data acquisition: FE, LM, MK, CF, AH, and AM. Data analysis and drafting of the manuscript: FE. Critically revising manuscript: FE, CF, PW, CS, J-CT, and AM. All authors contributed to the article and approved the submitted version.

## Conflict of Interest

FE reports a grant from Ludwig-Maximilians-University Munich and honoraria from Accuray outside the submitted work.

The remaining authors declare that the research was conducted in the absence of any commercial or financial relationships that could be construed as a potential conflict of interest.
